# Supplementary material for: Impact of Microbiota and Metabolites on Intestinal Integrity and Inflammation in Severe Obesity
Source: Pharmaceuticals (Basel). 2024 Jul 10;17(7):918. doi: 10.3390/ph17070918 (PMC11279642; doi:10.3390/ph17070918)
Supplement: Supplementary file 1 [file pharmaceuticals-17-00918-s001.zip › pharmaceuticals-3088226-supplementary.pdf]

Supplementary

Table S1. Differences in microbiota alpha diversity between intestinal inflammation and integrity groups.

variable	estimate	std.error	statistic	p.value
High vs low intestinal inflammation	4.67	2.54	1.84	0.0691
High vs low intestinal integrity	-0.184	2.51	-0.0736	0.942

Table S2. Differences in microbiota beta diversity between intestinal inflammation and integrity groups.

term	df	SumOfSqs	R2	statistic	p.value
High vs low intestinal inflammation	1	152	0.00988	0.838	0.905
High vs low intestinal integrity	1	168	0.0109	0.928	0.694

Table S3. Differences in metabolites between high and low intestinal inflammation groups.

variable	term	estimate	std.error	statistic	p.value
Propionic acid	high intestinal inflammation	-0.399	0.207	-1.92	0.058
TCDC	high intestinal inflammation	0.541	0.441	1.23	0.224
Iso valeric acid	high intestinal inflammation	-0.21	0.202	-1.04	0.301
UDC	high intestinal inflammation	-0.229	0.225	-1.02	0.311
CRP	high intestinal inflammation	-0.156	0.157	-0.996	0.322
GDC	high intestinal inflammation	0.287	0.325	0.883	0.38
TNFa	high intestinal inflammation	0.0582	0.0714	0.814	0.418
Haptoglobin	high intestinal inflammation	-0.0594	0.078	-0.761	0.449
Butyric acid	high intestinal inflammation	-0.219	0.291	-0.753	0.454
IL10	high intestinal inflammation	0.0635	0.0884	0.718	0.475
GCA	high intestinal inflammation	0.294	0.458	0.642	0.523
IL1b	high intestinal inflammation	4.72	7.99	0.59	0.557
CDC	high intestinal inflammation	-0.278	0.553	-0.504	0.616
IL4	high intestinal inflammation	-0.541	1.12	-0.482	0.631
IL6	high intestinal inflammation	-0.0888	0.198	-0.447	0.656
CA	high intestinal inflammation	-0.205	0.524	-0.39	0.697
IL8	high intestinal inflammation	0.0369	0.1	0.368	0.714
GCDC	high intestinal inflammation	0.103	0.298	0.346	0.73
DCA	high intestinal inflammation	-0.0593	0.235	-0.253	0.801
Iso butyric acid	high intestinal inflammation	0.104	0.537	0.194	0.847
GLC-3S	high intestinal inflammation	-0.0301	0.181	-0.167	0.868
Methyl butyric acid	high intestinal inflammation	0.0665	1.57	0.0424	0.966
LBP	high intestinal inflammation	0.0107	0.264	0.0405	0.968
Acetic acid	high intestinal inflammation	0.00161	0.0493	0.0327	0.974
SAA	high intestinal inflammation	0.000192	0.0747	0.00257	0.998

Table S4. Differences in gut bacteria between the high and low intestinal inflammatory groups.

asv	Base Mean	log2 Fold Change	lfcSE	stat	p.value	padj	label
ASV323	15.6	-24.9	2.51	-9.91	3.83E-23	1.16E-20	Bifidobacterium sp.
ASV261	8.32	24	2.52	9.52	1.69E-21	2.56E-19	Muribaculaceae sp.
ASV340	6.59	23.6	2.9	8.14	3.83E-16	3.87E-14	Hafnia-Obesumbacterium sp.
ASV163	6.25	23.5	2.9	8.1	5.51E-16	4.17E-14	Lachnospiraceae sp.
ASV12	1260	-1.42	0.483	-2.94	0.00328	0.199	Subdoligranulum sp.
ASV311	14.4	2.07	0.747	2.77	0.00561	0.283	UBA1819 sp.
ASV7	226	4.85	1.85	2.62	0.00886	0.384	Klebsiella sp.
ASV26	203	2.56	1.11	2.3	0.0213	0.806	Bacteroides dorei
ASV1	7000	-0.684	0.708	-0.966	0.334	0.972	Escherichia/Shigella sp.
ASV2	1780	-0.277	0.161	-1.72	0.0859	0.972	Blautia obeum
ASV3	1000	-0.6	0.503	-1.19	0.233	0.972	Streptococcus sp.
ASV4	2660	-0.887	0.668	-1.33	0.184	0.972	Bifidobacterium sp.
ASV5	1090	0.0423	0.133	0.319	0.75	0.972	Blautia massiliensis
ASV6	673	-0.503	0.648	-0.776	0.438	0.972	Bacteroides vulgatus
ASV8	1030	0.233	0.342	0.682	0.495	0.972	Fusicatenibacter saccharivorans
ASV9	533	-2.04	1.15	-1.76	0.0777	0.972	Akkermansia muciniphila
ASV10	855	0.151	0.347	0.434	0.664	0.972	Dorea longicatena
ASV11	1110	-0.474	0.743	-0.638	0.523	0.972	Ruminococcus bromii
ASV13	782	0.559	0.369	1.51	0.13	0.972	Faecalibacterium cf.
ASV14	758	-0.204	0.368	-0.554	0.58	0.972	Agathobacter sp.
ASV15	591	-0.26	0.323	-0.805	0.421	0.972	Blautia obeum
ASV17	452	0.216	0.31	0.698	0.485	0.972	Blautia faecis
ASV18	1650	-0.4	0.543	-0.737	0.461	0.972	Romboutsia ilealis
ASV19	625	0.45	0.472	0.953	0.34	0.972	Faecalibacterium prausnitzii
ASV20	62.6	-0.761	1.67	-0.456	0.649	0.972	Prevotella copri
ASV21	571	0.418	0.313	1.33	0.182	0.972	Lachnospiraceae sp.

ASV23	363	-0.152	0.347	-0.44	0.66	0.972	Anaerostipes hadrus
ASV24	29.6	0.827	1.22	0.68	0.496	0.972	Enterococcus sp.
ASV25	468	0.29	0.529	0.549	0.583	0.972	Agathobacter sp.
ASV27	13.8	-0.681	2.24	-0.304	0.761	0.972	Prevotella sp.
ASV28	338	-0.45	0.609	-0.739	0.46	0.972	Bifidobacterium breve
ASV30	276	0.362	0.516	0.701	0.483	0.972	Lachnospiraceae sp.
ASV31	146	-0.84	0.857	-0.98	0.327	0.972	Bacteroides uniformis
ASV32	14.7	2.77	2.89	0.959	0.338	0.972	Citrobacter sp.
ASV34	159	0.46	1.53	0.301	0.763	0.972	Holdemanella sp.
ASV37	290	0.305	0.462	0.66	0.509	0.972	Coprococcus comes
ASV38	157	-1.6	1.79	-0.898	0.369	0.972	Ruminococcus sp.
ASV39	211	0.221	0.478	0.463	0.644	0.972	Lachnospiraceae ND3007 group sp.
ASV42	89.2	0.353	0.818	0.432	0.666	0.972	Lachnospiraceae sp.
ASV43	1.11	-3.73	2.91	-1.28	0.2	0.972	Prevotella sp.
ASV44	155	-0.148	0.246	-0.601	0.548	0.972	Dorea formicigenerans
ASV46	246	0.471	0.456	1.03	0.301	0.972	Erysipelotrichaceae UCG-003 bacterium
ASV47	146	0.782	0.682	1.15	0.252	0.972	Bilophila wadsworthia
ASV48	109	2.06	2.01	1.02	0.306	0.972	Catenibacterium mitsuokai
ASV51	118	0.473	0.672	0.704	0.482	0.972	Faecalibacterium prausnitzii
ASV52	2.79	-2.46	2.9	-0.85	0.395	0.972	Akkermansia muciniphila
ASV53	187	-0.42	0.661	-0.636	0.525	0.972	Alistipes putredinis
ASV54	58.2	-0.61	0.767	-0.795	0.426	0.972	Roseburia sp.
ASV56	0.461	2.37	2.91	0.816	0.414	0.972	Megasphaera massiliensis
ASV57	140	1.23	0.935	1.32	0.187	0.972	Agathobacter sp.
ASV59	88.5	1.73	1.79	0.963	0.335	0.972	Bifidobacterium sp.
ASV61	1.19	1.52	2.9	0.524	0.6	0.972	Enterobacteriaceae sp.
ASV63	131	0.319	0.351	0.908	0.364	0.972	Monoglobus sp.
ASV65	40.2	1.67	1.06	1.59	0.113	0.972	Lachnoclostridium edouardi
ASV66	5.79	5.88	2.9	2.02	0.0429	0.972	Streptococcus sp.

ASV67	12.4	-2.59	2.89	-0.897	0.37	0.972	Megamonas funiformis
ASV69	44.8	-0.921	0.961	-0.958	0.338	0.972	NK4A214 group sp.
ASV71	33.6	0.702	0.952	0.737	0.461	0.972	Alistipes finegoldii
ASV72	77.6	-0.281	0.982	-0.286	0.775	0.972	Christensenellaceae R-7 group sp.
ASV73	199	0.473	0.768	0.615	0.538	0.972	Clostridium sensu stricto 1 sp.
ASV75	10.9	-2.12	2.89	-0.733	0.464	0.972	Holdemanella biformis
ASV76	44.6	1.54	1.42	1.09	0.277	0.972	Bacteroides dorei
ASV77	161	-0.435	1.55	-0.281	0.778	0.972	Holdemanella sp.
ASV79	58.6	0.324	0.72	0.45	0.653	0.972	Bacteroides sp.
ASV80	301	0.385	0.836	0.46	0.646	0.972	Intestinibacter bartlettii
ASV81	80.7	0.769	0.686	1.12	0.262	0.972	Roseburia inulinivorans
ASV85	77.4	-0.676	1.48	-0.458	0.647	0.972	Methanobrevibacter sp.
ASV86	65.1	-0.476	1.5	-0.317	0.751	0.972	Desulfovibrio sp.
ASV87	44.3	-0.722	1.35	-0.536	0.592	0.972	Blautia sp.
ASV88	76	-1.36	1.02	-1.34	0.181	0.972	Bacteroides sp.
ASV89	65.5	-0.364	0.607	-0.599	0.549	0.972	Butyricicoccus sp.
ASV90	83.2	-0.436	0.916	-0.476	0.634	0.972	Anaerostipes hadrus
ASV91	65.6	-0.654	0.987	-0.663	0.507	0.972	Blautia sp.
ASV92	1.24	3.7	2.91	1.28	0.202	0.972	Megasphaera cf.
ASV93	42.9	1.52	1.36	1.12	0.263	0.972	Coprococcus eutactus
ASV94	7.12	-3.14	2.36	-1.33	0.183	0.972	Phascolarctobacterium faecium
ASV96	35	-2.16	1.26	-1.72	0.0862	0.972	Bacteroides dorei
ASV97	8.89	-2.46	2.11	-1.16	0.245	0.972	Streptococcus sp.
ASV99	77.2	-0.232	0.538	-0.432	0.666	0.972	Incertae Sedis sp.
ASV100	63.2	0.337	0.623	0.541	0.588	0.972	Agathobacter sp.
ASV101	48.2	-1.08	0.807	-1.34	0.181	0.972	UCG-002 sp.
ASV102	44.1	0.578	1.25	0.461	0.644	0.972	Intestinimonas sp.
ASV103	54.9	0.758	0.62	1.22	0.222	0.972	Lachnospiraceae sp.
ASV105	62	0.208	0.468	0.445	0.656	0.972	Coprococcus catus
ASV106	62.5	1.69	1.58	1.07	0.285	0.972	Oscillospirales sp.
ASV107	4.11	-5.54	2.9	-1.91	0.0565	0.972	Escherichia/Shigella sp.
ASV109	46.4	2.54	1.44	1.76	0.078	0.972	Bacteroides cellulosilyticus
ASV112	48.9	-1.25	1.44	-0.864	0.388	0.972	Oscillospirales sp.

ASV114	56.8	-0.294	1.04	-0.283	0.777	0.972	Ruminococcus bicirculans
ASV115	30.5	-0.998	1.34	-0.743	0.458	0.972	Ruminococcus callidus
ASV116	99	-1.26	1.44	-0.877	0.38	0.972	Clostridium sensu stricto 1 sp.
ASV117	19.3	0.79	1.78	0.444	0.657	0.972	Phascolarctobacterium succinatutens
ASV120	38	0.308	1.02	0.302	0.763	0.972	Roseburia intestinalis
ASV121	47.2	1.15	0.708	1.62	0.104	0.972	Lachnoclostridium sp.
ASV122	5.23	-0.945	2.89	-0.327	0.744	0.972	Bacteroides eggerthii
ASV123	49.2	0.44	0.541	0.813	0.416	0.972	Lachnospiraceae FCS020 group sp.
ASV124	5.85	3.05	2.34	1.3	0.192	0.972	Sutterella massiliensis
ASV125	59.6	0.825	1.68	0.49	0.624	0.972	Faecalibacterium prausnitzii
ASV127	41.4	-0.138	0.499	-0.278	0.781	0.972	Marvinbryantia sp.
ASV128	26.2	-2.27	1.28	-1.78	0.0756	0.972	Lachnospiraceae sp.
ASV129	4.52	-5.67	2.9	-1.95	0.0508	0.972	Escherichia/Shigella sp.
ASV130	6.61	2.7	2.89	0.933	0.351	0.972	Desulfovibrio sp.
ASV131	13.9	1.2	1.12	1.07	0.285	0.972	Blautia sp.
ASV132	62.4	-0.297	1.18	-0.252	0.801	0.972	Oscillospirales sp.
ASV133	15.5	-1.25	1.6	-0.781	0.435	0.972	NK4A214 group sp.
ASV134	40.5	-0.409	0.755	-0.542	0.588	0.972	Faecalibacterium sp.
ASV135	1.54	4.02	2.9	1.38	0.167	0.972	Dialister sp.
ASV136	28.8	-0.789	1.99	-0.396	0.692	0.972	Blautia stercoris
ASV142	24.4	0.647	0.905	0.715	0.474	0.972	Butyricicoccus faecihominis
ASV144	11.8	-1.12	1.86	-0.603	0.546	0.972	Lachnospiraceae sp.
ASV145	36.5	1.48	0.991	1.5	0.134	0.972	Lachnospiraceae NK4A136 group sp.
ASV146	55.3	-0.538	1.56	-0.345	0.73	0.972	Bifidobacterium bifidum
ASV147	69.7	1.66	1.21	1.37	0.172	0.972	Bacteroides fragilis
ASV148	36.1	-0.435	0.582	-0.748	0.455	0.972	Blautia sp.
ASV149	35.2	-1.28	1.83	-0.699	0.485	0.972	Faecalibacterium prausnitzii
ASV150	32.4	3.14	2.13	1.47	0.141	0.972	Bacteroides coprocola
ASV151	33.5	1.27	0.779	1.62	0.104	0.972	env.OPS 17 sp.
ASV153	29.5	-0.94	1.21	-0.778	0.436	0.972	Christensenellaceae R-7 group sp.
ASV154	57.4	3.04	1.57	1.93	0.0535	0.972	Ruminococcus sp.
ASV155	15.5	-0.351	1.04	-0.338	0.735	0.972	Alistipes shahii
ASV156	24.9	0.477	1.49	0.32	0.749	0.972	Paraprevotella sp.

ASV157	3.95	5.34	2.51	2.12	0.0338	0.972	Acidaminococcus intestini
ASV159	18.6	-0.483	1.4	-0.345	0.73	0.972	Oscillospiraceae sp.
ASV160	52.7	0.353	0.831	0.425	0.671	0.972	Lachnospiraceae sp.
ASV162	82.8	-1.39	1	-1.38	0.166	0.972	Terrisporobacter mayombei
ASV164	20.3	-1.13	0.972	-1.17	0.244	0.972	UCG-005 sp.
ASV165	8.03	1.21	1.47	0.828	0.408	0.972	Roseburia sp.
ASV166	24.2	0.766	1.12	0.683	0.495	0.972	Butyricicoccus sp.
ASV171	25.3	0.792	0.753	1.05	0.293	0.972	Odoribacter splanchnicus
ASV172	0.622	-2.95	2.91	-1.01	0.311	0.972	Raoultella sp.
ASV173	22.8	3.29	1.79	1.84	0.0652	0.972	Ruminococcus sp.
ASV174	19.9	-0.932	1.28	-0.727	0.467	0.972	Lachnospira sp.
ASV175	27.4	0.618	0.868	0.712	0.476	0.972	Lactococcus garvieae
ASV176	31.2	0.666	0.948	0.702	0.483	0.972	Senegalimassilia anaerobia
ASV179	4.85	-5.77	2.9	-1.99	0.0468	0.972	Dialister succinatiphilus
ASV180	15.7	-0.425	0.859	-0.494	0.621	0.972	Lachnoclostridium sp.
ASV181	22.9	-0.458	1.27	-0.361	0.718	0.972	Lachnospira sp.
ASV184	11.2	-2.56	1.59	-1.61	0.107	0.972	Lachnospiraceae sp.
ASV186	59.8	-0.576	0.976	-0.59	0.555	0.972	Turicibacter sanguinis
ASV191	19.7	0.476	1.23	0.387	0.699	0.972	Lachnospiraceae sp.
ASV192	4.92	1.24	1.3	0.95	0.342	0.972	Lachnoclostridium sp.
ASV194	0.742	3	2.91	1.03	0.302	0.972	Bacteroides intestinalis
ASV195	5.98	5.92	2.9	2.04	0.0414	0.972	Mogibacterium sp.
ASV196	3.56	1.67	1.91	0.876	0.381	0.972	Barnesiella sp.
ASV197	16.7	-1.08	1.16	-0.93	0.353	0.972	UCG-002 sp.
ASV198	25.7	-0.496	0.379	-1.31	0.191	0.972	Lachnospiraceae sp.
ASV199	10	1	2.69	0.374	0.709	0.972	Bacteroides vulgatus
ASV200	17.1	0.93	0.634	1.47	0.142	0.972	Lachnospira pectinoschiza
ASV201	3.37	-0.727	2.85	-0.255	0.798	0.972	Barnesiella intestinihominis
ASV202	7.27	1.84	1.69	1.09	0.276	0.972	Lactobacillus sp.
ASV205	6.94	1.3	1.79	0.728	0.466	0.972	Blautia caecimuris
ASV206	1.82	4.25	2.9	1.46	0.144	0.972	Blautia sp.
ASV207	32.3	-1.15	0.771	-1.49	0.136	0.972	Lachnospiraceae sp.
ASV209	17.3	-0.285	0.79	-0.361	0.718	0.972	Alistipes obesi
ASV210	13.1	-0.983	0.879	-1.12	0.263	0.972	Marvinbryantia sp.

ASV212	19.5	-0.797	1.07	-0.742	0.458	0.972	Colidextribacter sp.
ASV213	25.3	1.11	1.97	0.564	0.573	0.972	Coprococcus sp.
ASV214	23.5	-0.828	0.811	-1.02	0.307	0.972	Christensenellaceae R-7 group sp.
ASV216	24.6	0.327	1.06	0.31	0.757	0.972	Lachnospiraceae FCS020 group bacterium
ASV217	26.3	0.5	0.989	0.505	0.613	0.972	Enterorhabdus sp.
ASV218	36.5	-0.424	1.58	-0.268	0.789	0.972	Oscillospiraceae sp.
ASV219	36.5	-0.615	1.94	-0.318	0.751	0.972	Romboutsia sedimentorum
ASV220	20.9	0.296	1.18	0.251	0.802	0.972	Lachnospiraceae NK4A136 group bacterium
ASV222	3.61	-3.23	2.72	-1.19	0.235	0.972	Barnesiella sp.
ASV224	1.06	-3.65	2.91	-1.26	0.209	0.972	Alistipes sp.
ASV226	0.902	2.76	2.91	0.95	0.342	0.972	Barnesiella sp.
ASV227	11.8	-1.15	2.89	-0.399	0.69	0.972	Barnesiella sp.
ASV228	2.94	4.92	2.9	1.69	0.0901	0.972	Bacteroides vulgatus
ASV232	6.78	0.67	1.46	0.46	0.645	0.972	Haemophilus sp.
ASV236	1.17	3.63	2.91	1.25	0.212	0.972	Bacteroides coprophilus
ASV237	0.221	-1.73	2.91	-0.595	0.552	0.972	Muribaculaceae sp.
ASV239	0.597	2.72	2.91	0.935	0.35	0.972	Morganella morganii
ASV241	11.6	-0.49	1.17	-0.419	0.676	0.972	UCG-002 sp.
ASV243	11.2	0.433	1.28	0.34	0.734	0.972	Lachnospiraceae sp.
ASV249	1.02	-0.987	2.9	-0.34	0.734	0.972	Prevotella sp.
ASV253	35	0.795	1.48	0.537	0.591	0.972	Turicibacter sanguinis
ASV257	14	0.613	1.8	0.341	0.733	0.972	Blautia sp.
ASV260	13.1	0.785	1.29	0.607	0.544	0.972	Alistipes inops
ASV262	2.56	-4.87	2.9	-1.68	0.0933	0.972	Parabacteroides distasonis
ASV263	14.1	0.933	1.36	0.688	0.492	0.972	Subdoligranulum sp.
ASV268	14.3	-1.03	1.26	-0.818	0.413	0.972	UCG-005 sp.
ASV270	3.95	1.94	2.89	0.669	0.503	0.972	Bifidobacterium animalis
ASV271	12.5	0.198	0.727	0.272	0.786	0.972	Lachnospiraceae sp.
ASV273	11.2	1.86	1.59	1.16	0.244	0.972	Negativibacillus sp.
ASV277	0.583	0.749	2.91	0.258	0.797	0.972	Barnesiella sp.
ASV278	1.44	-4.08	2.91	-1.41	0.16	0.972	Subdoligranulum sp.
ASV280	19	-1.39	1.94	-0.717	0.473	0.972	Clostridia UCG-014 sp.
ASV282	0.726	2.97	2.91	1.02	0.307	0.972	Solobacterium sp.
ASV283	12	-1.13	1.12	-1.02	0.31	0.972	Tyzzerella sp.
ASV287	4.49	1.13	2.89	0.39	0.696	0.972	Paraprevotella sp.
ASV288	1.16	1.29	2.9	0.443	0.658	0.972	Mitsuokella jalaludinii

ASV289	20.2	-1.03	0.663	-1.55	0.121	0.972	Family XIII AD3011 group sp.
ASV291	2.58	1.39	1.88	0.741	0.459	0.972	Streptococcus mutans
ASV292	14.8	0.797	1.83	0.435	0.663	0.972	Allisonella histaminiformans
ASV295	0.296	-0.789	2.91	-0.271	0.787	0.972	Blautia sp.
ASV297	5.65	-0.638	1.5	-0.424	0.672	0.972	Ruminococcaceae sp.
ASV298	0.91	3.28	2.91	1.13	0.259	0.972	Subdoligranulum sp.
ASV299	8.4	0.799	1.31	0.61	0.542	0.972	Lachnospiraceae UCG-001 sp.
ASV301	10	0.409	1.43	0.285	0.776	0.972	Agathobacter sp.
ASV302	9.17	-2.14	2.29	-0.937	0.349	0.972	Clostridia UCG-014 sp.
ASV303	5.51	0.711	1.62	0.439	0.661	0.972	Monoglobus pectinilyticus
ASV309	23.8	3.19	1.98	1.61	0.107	0.972	Oscillospirales sp.
ASV310	1.74	3.24	2.65	1.22	0.222	0.972	Lactobacillus sp.
ASV315	3.63	-1.59	2.89	-0.55	0.582	0.972	Oscillospirales sp.
ASV320	3.97	5.34	2.9	1.84	0.0657	0.972	Clostridia UCG-014 sp.
ASV321	0.618	-2.94	2.91	-1.01	0.313	0.972	Peptococcus sp.
ASV322	3.29	3.73	2.9	1.29	0.199	0.972	Bilophila wadsworthia
ASV328	7.52	0.496	1.75	0.284	0.777	0.972	Clostridia UCG-014 sp.
ASV329	0.295	1.87	2.91	0.642	0.521	0.972	Blautia sp.
ASV330	0.519	-2.72	2.91	-0.933	0.351	0.972	Lachnospiraceae sp.
ASV331	0.705	-3.09	2.91	-1.06	0.288	0.972	Oscillospirales sp.
ASV337	1.23	3.7	2.91	1.27	0.203	0.972	Bacteroides sp.
ASV345	0.492	-2.64	2.91	-0.908	0.364	0.972	Dialister succinatiphilus
ASV348	6.28	2.07	2.59	0.801	0.423	0.972	Blautia sp.
ASV351	0.648	-3	2.91	-1.03	0.302	0.972	Catenibacterium sp.
ASV352	9.22	-0.548	0.792	-0.692	0.489	0.972	Incertae Sedis sp.
ASV353	0.821	0.784	2.9	0.27	0.787	0.972	Subdoligranulum sp.
ASV356	3.7	-1.17	2.89	-0.405	0.685	0.972	Blautia luti
ASV359	0.573	1.12	2.91	0.384	0.701	0.972	Clostridia UCG-014 sp.
ASV360	1.07	-3.69	2.91	-1.27	0.205	0.972	Blautia sp.
ASV373	26.1	-0.278	0.915	-0.303	0.762	0.972	Incertae Sedis sp.
ASV374	5.69	1.14	2.11	0.542	0.588	0.972	Subdoligranulum sp.
ASV376	2.48	-4.83	2.9	-1.66	0.096	0.972	Barnesiella sp.
ASV380	0.65	2.82	2.91	0.97	0.332	0.972	Prevotella sp.
ASV396	1.15	3.61	2.91	1.24	0.214	0.972	Bilophila wadsworthia
ASV399	13.7	-0.681	0.905	-0.752	0.452	0.972	Alistipes ihumii
ASV409	0.751	-2.38	2.91	-0.817	0.414	0.972	Clostridia UCG-014 sp.
ASV412	0.467	-2.57	2.91	-0.885	0.376	0.972	Bilophila sp.
ASV416	7.26	0.392	0.856	0.458	0.647	0.972	Oscillibacter sp.
ASV422	1.26	-1.47	2.1	-0.701	0.484	0.972	Lactobacillus delbrueckii

ASV426	7.4	-0.651	1.59	-0.409	0.682	0.972	Clostridia UCG-014 sp.
ASV428	5.8	-0.687	1.53	-0.45	0.653	0.972	Lachnospiraceae sp.
ASV429	0.874	-1.46	2.9	-0.503	0.615	0.972	Ruminococcus sp.
ASV436	0.36	2.09	2.91	0.717	0.473	0.972	Bacteroides sp.
ASV448	1.73	-1.63	2.78	-0.585	0.559	0.972	Clostridia UCG-014 sp.
ASV452	4.1	-4.01	2.9	-1.38	0.166	0.972	Enterococcus sp.
ASV481	8.51	0.689	1.61	0.428	0.669	0.972	Lachnospiraceae sp.
ASV483	6.33	-0.763	1.48	-0.514	0.607	0.972	Eggerthella lenta
ASV490	1.05	-3.65	2.91	-1.25	0.21	0.972	Lachnospiraceae sp.
ASV528	2.16	-1.09	2.33	-0.468	0.64	0.972	Alistipes massiliensis
ASV548	9.36	-1.27	1.14	-1.11	0.266	0.972	Christensenellaceae R-7 group sp.
ASV571	1.14	2.07	2.9	0.713	0.476	0.972	Clostridium sensu stricto 1 disporicum
ASV581	0.637	1.34	2.91	0.461	0.645	0.972	Lactobacillus sp.
ASV626	0.897	-3.44	2.91	-1.18	0.237	0.972	Clostridium sensu stricto 1 tertium
ASV646	1.4	3.87	2.9	1.33	0.183	0.972	Lactobacillus sp.
ASV655	1.63	-2.09	2.9	-0.722	0.47	0.972	Clostridium sensu stricto 1 sp.
ASV669	1.1	3.54	2.91	1.22	0.223	0.972	Lachnospiraceae sp.
ASV762	1.14	3.59	2.91	1.23	0.217	0.972	Agathobacter sp.
ASV867	0.536	-1.54	2.91	-0.531	0.596	0.972	Sarcina ventriculi
ASV1047	0.627	-2.95	2.91	-1.02	0.31	0.972	Agathobacter sp.
ASV544	1.46	-0.711	2.9	-0.245	0.806	0.973	Romboutsia sp.
ASV29	188	0.184	0.851	0.216	0.829	0.973	Bacteroides uniformis
ASV33	329	-0.119	0.532	-0.223	0.823	0.973	Lachnospiraceae sp.
ASV40	193	-0.0703	0.371	-0.189	0.85	0.973	Blautia sp.
ASV68	7.54	0.241	1.32	0.183	0.855	0.973	Veillonella atypica
ASV143	60.1	0.388	1.81	0.214	0.83	0.973	Ruminococcus sp.
ASV169	4.17	0.382	2.24	0.17	0.865	0.973	UCG-002 sp.
ASV177	3.26	-0.349	2.09	-0.167	0.867	0.973	NK4A214 group sp.
ASV185	9.14	0.356	1.68	0.212	0.832	0.973	Roseburia hominis
ASV223	3.07	-0.484	2.85	-0.17	0.865	0.973	Coprococcus eutactus
ASV231	0.449	-0.542	2.91	-0.186	0.852	0.973	Muribaculaceae sp.
ASV251	4.03	-0.486	2.89	-0.168	0.866	0.973	Bacteroides sp.
ASV294	14.4	-0.329	2.13	-0.155	0.877	0.973	Parabacteroides johnsonii
ASV317	10.4	0.221	0.993	0.223	0.824	0.973	Lachnospiraceae sp.
ASV363	3.6	-0.641	2.84	-0.226	0.821	0.973	Lachnospiraceae sp.
ASV379	5.01	-0.311	1.41	-0.22	0.826	0.973	Coriobacteriales Incertae Sedis sp.

ASV402	11.7	-0.42	2.36	-0.178	0.859	0.973	Bacteroides ovatus
ASV406	2.7	-0.453	2.89	-0.157	0.876	0.973	Lachnospiraceae sp.
ASV437	11.9	-0.254	1.19	-0.213	0.831	0.973	Lachnospiraceae sp.
ASV441	0.527	-0.529	2.91	-0.182	0.856	0.973	Blautia glucerasea
ASV464	7.32	-0.236	1.14	-0.208	0.836	0.973	Slackia isoflavoniconvertens
ASV493	4.04	-0.565	2.84	-0.199	0.843	0.973	Clostridia UCG-014 sp.
ASV660	8.92	0.437	2.75	0.159	0.874	0.973	Leuconostoc sp.
ASV78	107	-0.133	0.999	-0.134	0.894	0.988	UCG-002 sp.
ASV16	840	-0.0321	0.369	-0.0871	0.931	0.988	Subdoligranulum sp.
ASV22	473	0.0412	0.406	0.101	0.919	0.988	Collinsella aerofaciens
ASV35	273	0.122	1.37	0.089	0.929	0.988	Dialister invisus
ASV41	499	-0.0588	0.742	-0.0792	0.937	0.988	Clostridium sensu stricto 1 celatum
ASV49	113	-0.0593	0.825	-0.072	0.943	0.988	Bacteroides caccae
ASV58	0.76	0.242	2.91	0.0831	0.934	0.988	Akkermansia sp.
ASV60	66.2	0.0604	0.714	0.0846	0.933	0.988	Bacteroides sp.
ASV104	89.5	0.0888	0.818	0.109	0.914	0.988	Eggerthellaceae sp.
ASV188	21.3	-0.243	2.16	-0.112	0.91	0.988	Oscillospirales sp.
ASV203	3.76	-0.222	2.89	-0.0767	0.939	0.988	Desulfovibrio piger
ASV244	18.8	0.125	1.48	0.0845	0.933	0.988	Ruminococcaceae sp.
ASV332	0.36	0.373	2.91	0.128	0.898	0.988	Lachnospiraceae sp.
ASV339	9.27	-0.114	1.18	-0.0966	0.923	0.988	Lachnospiraceae sp.
ASV413	1.56	-0.211	2.9	-0.0728	0.942	0.988	Faecalibacterium sp.
ASV432	2.28	-0.322	2.89	-0.111	0.911	0.988	UCG-008 sp.
ASV566	0.047	0.193	2.92	0.0661	0.947	0.99	Barnesiella sp.
ASV36	1.88	0.0282	2.34	0.0121	0.99	0.995	Clostridium sensu stricto 1 perfringens
ASV45	150	-0.00605	0.648	-0.00933	0.993	0.995	Parabacteroides merdae
ASV50	46.6	0.0584	1.34	0.0435	0.965	0.995	Bacteroides massiliensis
ASV62	64.8	-0.0297	0.74	-0.0401	0.968	0.995	CAG-56 sp.
ASV74	73	-0.0222	1.06	-0.0209	0.983	0.995	Lachnospiraceae sp.
ASV111	60.3	0.00851	0.743	0.0115	0.991	0.995	Subdoligranulum sp.
ASV119	40.4	-0.0333	0.977	-0.0341	0.973	0.995	Parasutterella excrementihominis

ASV168	16.6	0.0269	1.31	0.0205	0.984	0.995	Alistipes finegoldii
ASV255	5.7	-0.0176	2.89	-0.0061	0.995	0.995	Peptococcus sp.
ASV341	16.4	-0.0221	1.15	-0.0193	0.985	0.995	Howardella ureilytica
ASV354	5.16	0.0648	2.28	0.0284	0.977	0.995	Clostridia sp.
ASV375	3.75	-0.119	2.42	-0.0493	0.961	0.995	Bacteroides clarus
ASV813	0.306	0.071	2.91	0.0244	0.981	0.995	Ruminococcus sp.

Table S5. Differences in metabolites between high and low intestinal inflammation groups.

variable	term	estimate	std.error	statistic	p.value
CA	high intestinal integrity	0.902	0.534	1.69	0.0952
CDC	high intestinal integrity	0.518	0.564	0.918	0.361
DCA	high intestinal integrity	-0.135	0.245	-0.553	0.582
GCA	high intestinal integrity	0.516	0.482	1.07	0.288
GCDC	high intestinal integrity	0.335	0.314	1.07	0.29
GDC	high intestinal integrity	0.316	0.345	0.918	0.361
GLC-3S	high intestinal integrity	-0.0227	0.199	-0.114	0.91
TCDC	high intestinal integrity	1.09	0.457	2.38	0.0194
UDC	high intestinal integrity	-0.19	0.237	-0.804	0.424
CRP	high intestinal integrity	0.00326	0.161	0.0203	0.984
Haptoglobin	high intestinal integrity	-0.0407	0.0828	-0.492	0.624
SAA	high intestinal integrity	-0.048	0.0782	-0.614	0.541
LBP	high intestinal integrity	-0.434	0.262	-1.66	0.101
IL1b	high intestinal integrity	-9.66	8.37	-1.15	0.252
IL6	high intestinal integrity	0.194	0.209	0.927	0.356
TNFa	high intestinal integrity	0.0914	0.0717	1.27	0.206
IL4	high intestinal integrity	-0.365	1.19	-0.307	0.76
IL10	high intestinal integrity	-0.00569	0.0934	-0.061	0.952
IL8	high intestinal integrity	0.266	0.102	2.6	0.011
Acetic acid	high intestinal integrity	-0.104	0.0503	-2.06	0.0425
Propionic acid	high intestinal integrity	-0.00023	0.222	-0.00105	0.999
Butyric acid	high intestinal integrity	-0.329	0.305	-1.08	0.283
Iso butyric acid	high intestinal integrity	-0.586	0.561	-1.04	0.299
Methyl butyric acid	high intestinal integrity	-1.39	1.68	-0.828	0.41
Iso valeric acid	high intestinal integrity	-0.591	0.196	-3.02	0.00335

Table S6. Differences in gut bacteria between the high and low intestinal integrity groups.

asv	Base Mean	log2 Fold Change	lfcSE	stat	p.value	padj	label
ASV199	10	-22.7	2.44	-9.29	1.54E-20	4.61E-18	Bacteroides vulgatus
ASV340	4.08	23.6	2.95	7.98	1.47E-15	2.21E-13	Hafnia-Obesumbacterium sp.
ASV119	62.4	-3.27	0.97	-3.38	0.000737	0.0737	Parasutterella excrementihominis
ASV72	77.6	-2.56	0.959	-2.67	0.00754	0.566	Christensenellaceae R-7 group sp.
ASV1	7540	-1.77	0.722	-2.45	0.0143	0.677	Escherichia/Shigella sp.
ASV23	490	-0.972	0.393	-2.47	0.0134	0.677	Anaerostipes hadrus
ASV59	141	-4.14	1.75	-2.36	0.0181	0.677	Bifidobacterium sp.
ASV71	50	2.33	0.972	2.4	0.0163	0.677	Alistipes finegoldii
ASV2	1780	-0.334	0.162	-2.05	0.0399	0.759	Blautia obeum
ASV17	452	-0.606	0.31	-1.95	0.0507	0.759	Blautia faecis
ASV21	571	-0.606	0.316	-1.92	0.0552	0.759	Lachnospiraceae sp.
ASV26	275	-2.27	1.18	-1.92	0.0544	0.759	Bacteroides dorei
ASV66	5.79	-5.55	2.96	-1.88	0.0607	0.759	Streptococcus sp.
ASV79	73.7	-1.4	0.746	-1.88	0.0605	0.759	Bacteroides sp.
ASV128	26.2	2.64	1.28	2.05	0.0402	0.759	Lachnospiraceae sp.
ASV165	44	3.14	1.43	2.2	0.0281	0.759	Roseburia sp.
ASV179	4.02	5.83	2.95	1.97	0.0484	0.759	Dialister succinatiphilus
ASV186	79.8	1.93	0.995	1.94	0.0524	0.759	Turicibacter sanguinis
ASV209	27.4	1.64	0.839	1.95	0.0513	0.759	Alistipes obesi
ASV222	5.46	-5.46	2.41	-2.27	0.0234	0.759	Barnesiella sp.
ASV231	6.28	-5.66	2.96	-1.91	0.0556	0.759	Muribaculaceae sp.
ASV406	8.21	4.72	2.47	1.91	0.056	0.759	Lachnospiraceae sp.
ASV452	6.39	-5.68	2.96	-1.92	0.0545	0.759	Enterococcus sp.
ASV493	5.94	-5.57	2.67	-2.08	0.0371	0.759	Clostridia UCG-014 sp.
ASV30	276	-0.926	0.518	-1.79	0.074	0.889	Lachnospiraceae sp.
ASV13	782	-0.619	0.375	-1.65	0.0988	0.928	Faecalibacterium cf.
ASV50	38.3	-2.2	1.32	-1.67	0.095	0.928	Bacteroides massiliensis
ASV52	3.61	-4.88	2.96	-1.65	0.0989	0.928	Akkermansia muciniphila
ASV68	7.54	-2.07	1.3	-1.59	0.111	0.928	Veillonella atypica
ASV114	56.8	-1.7	1.04	-1.64	0.101	0.928	Ruminococcus bicirculans
ASV147	69.7	2.07	1.22	1.7	0.0899	0.928	Bacteroides fragilis
ASV175	36.8	-1.46	0.902	-1.62	0.105	0.928	Lactococcus garvieae
ASV202	7.34	-3.08	1.77	-1.74	0.0818	0.928	Lactobacillus sp.
ASV232	9.26	-2.28	1.42	-1.61	0.108	0.928	Haemophilus sp.

ASV303	7.48	2.88	1.68	1.71	0.0866	0.928	Monoglobus pectinilyticus
ASV329	3.2	-4.71	2.96	-1.59	0.111	0.928	Blautia sp.
ASV3	1000	-0.22	0.514	-0.428	0.669	0.985	Streptococcus sp.
ASV4	2660	-0.838	0.68	-1.23	0.218	0.985	Bifidobacterium sp.
ASV5	1090	0.129	0.134	0.962	0.336	0.985	Blautia massiliensis
ASV6	595	-0.129	0.65	-0.198	0.843	0.985	Bacteroides vulgatus
ASV7	154	-0.398	2.1	-0.19	0.85	0.985	Klebsiella sp.
ASV8	1030	-0.124	0.348	-0.355	0.723	0.985	Fusicatenibacter saccharivorans
ASV9	533	-1.38	1.19	-1.16	0.248	0.985	Akkermansia muciniphila
ASV10	855	-0.186	0.352	-0.529	0.597	0.985	Dorea longicatena
ASV11	1110	0.176	0.757	0.232	0.816	0.985	Ruminococcus bromii
ASV12	1260	-0.413	0.507	-0.815	0.415	0.985	Subdoligranulum sp.
ASV14	758	-0.0984	0.375	-0.263	0.793	0.985	Agathobacter sp.
ASV15	591	-0.214	0.328	-0.651	0.515	0.985	Blautia obeum
ASV16	840	0.129	0.375	0.345	0.73	0.985	Subdoligranulum sp.
ASV18	1650	-0.196	0.553	-0.355	0.723	0.985	Romboutsia ilealis
ASV19	625	-0.568	0.479	-1.19	0.236	0.985	Faecalibacterium prausnitzii
ASV20	62.6	-1.75	1.68	-1.04	0.299	0.985	Prevotella copri
ASV22	473	-0.28	0.412	-0.679	0.497	0.985	Collinsella aerofaciens
ASV24	29.6	0.165	1.24	0.133	0.894	0.985	Enterococcus sp.
ASV25	468	-0.51	0.536	-0.951	0.341	0.985	Agathobacter sp.
ASV27	16.7	2.32	2.12	1.09	0.274	0.985	Prevotella sp.

ASV28	338	0.467	0.619	0.754	0.451	0.985	Bifidobacterium breve
ASV29	196	-0.723	0.87	-0.831	0.406	0.985	Bacteroides uniformis
ASV31	146	0.263	0.877	0.301	0.764	0.985	Bacteroides uniformis
ASV32	14.7	3.5	2.89	1.21	0.226	0.985	Citrobacter sp.
ASV33	329	-0.0564	0.541	-0.104	0.917	0.985	Lachnospiraceae sp.
ASV34	159	0.533	1.56	0.342	0.732	0.985	Holdemanella sp.
ASV35	273	0.332	1.39	0.238	0.811	0.985	Dialister invisus
ASV36	1.2	-0.688	2.52	-0.273	0.785	0.985	Clostridium sensu stricto 1 perfringens
ASV37	290	0.0419	0.471	0.0889	0.929	0.985	Coprococcus comes
ASV38	130	-0.625	1.88	-0.332	0.74	0.985	Ruminococcus sp.
ASV39	211	-0.314	0.485	-0.647	0.518	0.985	Lachnospiraceae ND3007 group sp.
ASV40	193	-0.0399	0.377	-0.106	0.916	0.985	Blautia sp.
ASV41	499	0.278	0.754	0.369	0.712	0.985	Clostridium sensu stricto 1 celatum
ASV42	89.2	0.978	0.824	1.19	0.235	0.985	Lachnospiraceae sp.
ASV43	1.11	-3.24	2.96	-1.1	0.273	0.985	Prevotella sp.
ASV44	155	-0.222	0.249	-0.892	0.372	0.985	Dorea formicigenerans
ASV45	150	-0.317	0.658	-0.481	0.63	0.985	Parabacteroides merdae
ASV46	246	0.118	0.465	0.254	0.799	0.985	Erysipelotrichaceae UCG-003 bacterium
ASV47	147	0.407	0.695	0.585	0.559	0.985	Bilophila wadsworthia
ASV49	113	1.12	0.828	1.35	0.177	0.985	Bacteroides caccae
ASV51	118	-0.226	0.685	-0.33	0.741	0.985	Faecalibacterium prausnitzii
ASV53	187	0.641	0.67	0.956	0.339	0.985	Alistipes putredinis

ASV54	58.2	-0.534	0.781	-0.684	0.494	0.985	Roseburia sp.
ASV56	2.72	-4.48	2.96	-1.52	0.129	0.985	Megasphaera massiliensis
ASV57	140	-1.07	0.955	-1.12	0.265	0.985	Agathobacter sp.
ASV58	0.76	2.94	2.96	0.995	0.32	0.985	Akkermansia sp.
ASV60	66.2	-0.592	0.724	-0.819	0.413	0.985	Bacteroides sp.
ASV61	1.19	2.07	2.95	0.702	0.483	0.985	Enterobacteriaceae sp.
ASV62	64.8	0.185	0.752	0.246	0.806	0.985	CAG-56 sp.
ASV63	131	0.319	0.356	0.895	0.371	0.985	Monoglobus sp.
ASV65	40.2	-1.61	1.08	-1.49	0.135	0.985	Lachnoclostridium edouardi
ASV67	28.2	1.25	2.94	0.427	0.67	0.985	Megamonas funiformis
ASV69	44.8	-1.37	0.97	-1.41	0.158	0.985	NK4A214 group sp.
ASV73	199	0.435	0.782	0.557	0.577	0.985	Clostridium sensu stricto 1 sp.
ASV74	73	-0.19	1.08	-0.175	0.861	0.985	Lachnospiraceae sp.
ASV75	6.41	-1.48	2.94	-0.502	0.616	0.985	Holdemanella biformis
ASV76	38	-2.02	1.46	-1.39	0.165	0.985	Bacteroides dorei
ASV77	161	-0.18	1.58	-0.114	0.909	0.985	Holdemanella sp.
ASV78	107	0.723	1.01	0.714	0.475	0.985	UCG-002 sp.
ASV80	301	0.438	0.85	0.515	0.606	0.985	Intestinibacter bartlettii
ASV81	80.7	-0.581	0.7	-0.83	0.407	0.985	Roseburia inulinivorans
ASV85	77.4	0.116	1.51	0.077	0.939	0.985	Methanobrevibacter sp.
ASV86	84.3	-0.705	1.55	-0.455	0.649	0.985	Desulfovibrio sp.
ASV87	44.3	-0.235	1.37	-0.171	0.864	0.985	Blautia sp.
ASV88	86.6	0.604	1.06	0.572	0.567	0.985	Bacteroides sp.

ASV89	65.5	-0.535	0.615	-0.87	0.384	0.985	Butyricicoccus sp.
ASV90	83.2	-0.859	0.928	-0.925	0.355	0.985	Anaerostipes hadrus
ASV91	65.6	0.322	1.01	0.32	0.749	0.985	Blautia sp.
ASV92	1.24	-1.86	2.96	-0.631	0.528	0.985	Megasphaera cf.
ASV93	51.9	1.78	1.39	1.28	0.202	0.985	Coprococcus eutactus
ASV94	5.79	-1.94	2.27	-0.856	0.392	0.985	Phascolarctobacterium faecium
ASV96	13.3	-0.547	1.2	-0.455	0.649	0.985	Bacteroides dorei
ASV97	8.89	-3.06	2.14	-1.43	0.152	0.985	Streptococcus sp.
ASV99	77.2	-0.37	0.547	-0.677	0.498	0.985	Incertae Sedis sp.
ASV100	63.2	0.226	0.634	0.357	0.721	0.985	Agathobacter sp.
ASV101	48.2	0.738	0.825	0.894	0.371	0.985	UCG-002 sp.
ASV102	44.1	0.139	1.28	0.109	0.913	0.985	Intestinimonas sp.
ASV103	54.9	0.92	0.628	1.47	0.143	0.985	Lachnospiraceae sp.
ASV104	89.5	0.183	0.831	0.221	0.825	0.985	Eggerthellaceae sp.
ASV105	62	0.164	0.475	0.345	0.73	0.985	Coprococcus catus
ASV106	64	-1.38	1.67	-0.826	0.409	0.985	Oscillospirales sp.
ASV109	48.8	-1.41	1.51	-0.937	0.349	0.985	Bacteroides cellulosilyticus
ASV111	60.3	-0.0768	0.755	-0.102	0.919	0.985	Subdoligranulum sp.
ASV115	18.9	-0.781	1.32	-0.59	0.555	0.985	Ruminococcus callidus
ASV116	67	-0.104	1.44	-0.0722	0.942	0.985	Clostridium sensu stricto 1 sp.
ASV117	20.9	-2.1	1.87	-1.12	0.263	0.985	Phascolarctobacterium succinatutens
ASV120	38	0.169	1.04	0.163	0.871	0.985	Roseburia intestinalis
ASV121	47.2	-0.604	0.728	-0.83	0.407	0.985	Lachnoclostridium sp.
ASV122	6.02	-2.53	2.95	-0.86	0.39	0.985	Bacteroides eggerthii

ASV123	49.2	0.319	0.551	0.579	0.563	0.985	Lachnospiraceae FCS020 group sp.
ASV124	8.29	-0.498	2.21	-0.225	0.822	0.985	Sutterella massiliensis
ASV125	59.6	-1.29	1.71	-0.756	0.45	0.985	Faecalibacterium prausnitzii
ASV127	41.4	-0.416	0.506	-0.823	0.41	0.985	Marvinbryantia sp.
ASV130	1.9	0.885	2.95	0.3	0.764	0.985	Desulfovibrio sp.
ASV131	10.3	-0.761	1.11	-0.685	0.493	0.985	Blautia sp.
ASV132	62.4	-0.239	1.2	-0.199	0.842	0.985	Oscillospirales sp.
ASV133	15.5	-1.01	1.64	-0.618	0.536	0.985	NK4A214 group sp.
ASV134	40.5	-0.469	0.768	-0.611	0.541	0.985	Faecalibacterium sp.
ASV135	8.05	0.863	2.94	0.293	0.769	0.985	Dialister sp.
ASV136	21.4	0.118	2.09	0.0565	0.955	0.985	Blautia stercoris
ASV142	24.4	0.274	0.923	0.297	0.767	0.985	Butyricicoccus faecihominis
ASV143	60.1	0.772	1.84	0.419	0.675	0.985	Ruminococcus sp.
ASV144	11.8	0.664	1.9	0.35	0.726	0.985	Lachnospiraceae sp.
ASV145	36.5	1.15	1.01	1.13	0.256	0.985	Lachnospiraceae NK4A136 group sp.
ASV146	55.3	-0.68	1.59	-0.429	0.668	0.985	Bifidobacterium bifidum
ASV148	36.1	-0.036	0.594	-0.0606	0.952	0.985	Blautia sp.
ASV149	17.6	-0.467	2.05	-0.227	0.82	0.985	Faecalibacterium prausnitzii
ASV150	28.5	-0.376	2.25	-0.168	0.867	0.985	Bacteroides coprocola
ASV151	24.6	0.218	0.767	0.284	0.776	0.985	env.OPS 17 sp.

ASV153	29.5	-0.354	1.24	-0.287	0.774	0.985	Christensenellaceae R-7 group sp.
ASV154	42.6	-0.781	1.64	-0.476	0.634	0.985	Ruminococcus sp.
ASV155	15.9	-1.57	1.04	-1.51	0.131	0.985	Alistipes shahii
ASV156	24.9	-1.25	1.51	-0.83	0.406	0.985	Paraprevotella sp.
ASV157	2.67	-0.923	2.95	-0.313	0.754	0.985	Acidaminococcus intestini
ASV159	18.6	-0.44	1.43	-0.309	0.757	0.985	Oscillospiraceae sp.
ASV160	52.7	0.69	0.841	0.821	0.412	0.985	Lachnospiraceae sp.
ASV162	63.3	-0.15	1.01	-0.149	0.881	0.985	Terrisporobacter mayombei
ASV164	20.3	-0.361	1	-0.361	0.718	0.985	UCG-005 sp.
ASV166	24.2	-0.787	1.14	-0.69	0.49	0.985	Butyricicoccus sp.
ASV168	19.5	1.16	1.3	0.89	0.374	0.985	Alistipes finegoldii
ASV169	4.17	0.724	2.27	0.319	0.75	0.985	UCG-002 sp.
ASV171	25.3	-0.214	0.771	-0.277	0.781	0.985	Odoribacter splanchnicus
ASV172	0.622	-2.47	2.96	-0.833	0.405	0.985	Raoultella sp.
ASV173	12.2	-0.129	2.03	-0.0637	0.949	0.985	Ruminococcus sp.
ASV176	31.2	0.485	0.966	0.503	0.615	0.985	Senegalimassilia anaerobia
ASV177	3.26	0.146	2.12	0.0686	0.945	0.985	NK4A214 group sp.
ASV178	1.4	4.35	2.95	1.47	0.141	0.985	Prevotellaceae NK3B31 group sp.
ASV180	15.7	0.115	0.875	0.131	0.896	0.985	Lachnoclostridium sp.
ASV181	22.9	-0.627	1.29	-0.486	0.627	0.985	Lachnospira sp.
ASV184	11.2	-0.679	1.68	-0.404	0.686	0.985	Lachnospiraceae sp.

ASV185	9.14	-0.322	1.71	-0.189	0.85	0.985	Roseburia hominis
ASV188	21.3	1.41	2.18	0.646	0.518	0.985	Oscillospirales sp.
ASV189	0.376	-1.84	2.96	-0.619	0.536	0.985	Ruminococcus sp.
ASV191	19.7	0.354	1.25	0.283	0.777	0.985	Lachnospiraceae sp.
ASV192	2.79	-1.3	1.28	-1.02	0.31	0.985	Lachnoclostridium sp.
ASV194	2.76	-4.5	2.96	-1.52	0.128	0.985	Bacteroides intestinalis
ASV195	5.29	1.36	2.94	0.463	0.643	0.985	Mogibacterium sp.
ASV196	3.56	-1.85	1.94	-0.956	0.339	0.985	Barnesiella sp.
ASV197	16.7	0.983	1.18	0.829	0.407	0.985	UCG-002 sp.
ASV198	25.7	-0.103	0.389	-0.265	0.791	0.985	Lachnospiraceae sp.
ASV200	21.9	-0.775	0.683	-1.14	0.256	0.985	Lachnospira pectinoschiza
ASV201	7.21	-2.59	2.89	-0.895	0.371	0.985	Barnesiella intestinihominis
ASV203	3.76	-2.36	2.95	-0.801	0.423	0.985	Desulfovibrio piger
ASV205	9.76	-2.31	1.75	-1.32	0.186	0.985	Blautia caecimuris
ASV206	1.82	2.17	2.95	0.737	0.461	0.985	Blautia sp.
ASV207	32.3	-0.388	0.795	-0.487	0.626	0.985	Lachnospiraceae sp.
ASV210	13.1	-0.684	0.899	-0.761	0.447	0.985	Marvinbryantia sp.
ASV212	19.5	-0.245	1.1	-0.223	0.823	0.985	Colidextribacter sp.
ASV213	34.1	-1.43	1.95	-0.734	0.463	0.985	Coprococcus sp.
ASV214	23.5	-0.169	0.831	-0.203	0.839	0.985	Christensenellaceae R-7 group sp.

ASV216	24.6	1.2	1.06	1.13	0.258	0.985	Lachnospiraceae FCS020 group bacterium
ASV217	26.3	0.733	1	0.731	0.465	0.985	Enterorhabdus sp.
ASV218	36.5	1.26	1.6	0.789	0.43	0.985	Oscillospiraceae sp.
ASV219	61	2.18	1.84	1.19	0.234	0.985	Romboutsia sedimentorum
ASV220	20.9	0.659	1.2	0.551	0.582	0.985	Lachnospiraceae NK4A136 group bacterium
ASV223	3.07	0.169	2.9	0.0582	0.954	0.985	Coprococcus eutactus
ASV224	1.06	3.95	2.96	1.34	0.181	0.985	Alistipes sp.
ASV226	0.902	0.465	2.95	0.157	0.875	0.985	Barnesiella sp.
ASV227	25.1	-3.88	2.94	-1.32	0.187	0.985	Barnesiella sp.
ASV228	2.94	3.11	2.95	1.05	0.292	0.985	Bacteroides vulgatus
ASV236	0.281	2.25	2.96	0.761	0.447	0.985	Bacteroides coprophilus
ASV241	11.6	0.633	1.19	0.533	0.594	0.985	UCG-002 sp.
ASV243	11.2	1.32	1.28	1.04	0.301	0.985	Lachnospiraceae sp.
ASV244	25.6	0.938	1.49	0.63	0.529	0.985	Ruminococcaceae sp.
ASV249	0.375	2.58	2.96	0.872	0.383	0.985	Prevotella sp.
ASV251	5.3	-0.538	2.94	-0.183	0.855	0.985	Bacteroides sp.
ASV253	35	1.46	1.49	0.979	0.328	0.985	Turicibacter sanguinis
ASV255	5.7	-2.18	2.94	-0.742	0.458	0.985	Peptococcus sp.
ASV257	17.3	0.49	1.78	0.276	0.783	0.985	Blautia sp.
ASV260	21.2	1.43	1.35	1.06	0.289	0.985	Alistipes inops
ASV261	1.09	-2.07	2.96	-0.699	0.484	0.985	Muribaculaceae sp.
ASV262	0.698	-2.61	2.96	-0.882	0.378	0.985	Parabacteroides distasonis
ASV263	14.1	-0.553	1.39	-0.399	0.69	0.985	Subdoligranulum sp.
ASV268	14.3	0.369	1.29	0.286	0.775	0.985	UCG-005 sp.

ASV270	3.57	-0.354	2.89	-0.123	0.902	0.985	Bifidobacterium animalis
ASV271	12.5	0.147	0.738	0.199	0.843	0.985	Lachnospiraceae sp.
ASV273	11.2	-1.13	1.65	-0.686	0.493	0.985	Negativibacillus sp.
ASV277	0.583	1.27	2.96	0.428	0.669	0.985	Barnesiella sp.
ASV278	1.44	-0.497	2.95	-0.168	0.866	0.985	Subdoligranulum sp.
ASV280	22.7	-2.29	1.88	-1.22	0.224	0.985	Clostridia UCG-014 sp.
ASV282	1.36	-3.52	2.96	-1.19	0.234	0.985	Solobacterium sp.
ASV283	14.4	-0.141	1.16	-0.121	0.904	0.985	Tyzzerella sp.
ASV287	17	0.949	2.61	0.364	0.716	0.985	Paraprevotella sp.
ASV288	1.16	-0.798	2.95	-0.27	0.787	0.985	Mitsuokella jalaludinii
ASV289	20.2	-0.0818	0.685	-0.12	0.905	0.985	Family XIII AD3011 group sp.
ASV291	2.58	-2.34	1.88	-1.25	0.212	0.985	Streptococcus mutans
ASV292	14.8	0.0997	1.87	0.0533	0.957	0.985	Allisonella histaminiformans
ASV294	5.94	-0.963	2.69	-0.358	0.72	0.985	Parabacteroides johnsonii
ASV295	0.702	-0.591	2.96	-0.2	0.842	0.985	Blautia sp.
ASV297	5.65	1.87	1.49	1.25	0.21	0.985	Ruminococcaceae sp.
ASV298	0.878	3.7	2.96	1.25	0.211	0.985	Subdoligranulum sp.
ASV299	8.4	-0.287	1.34	-0.215	0.83	0.985	Lachnospiraceae UCG-001 sp.
ASV301	10	0.728	1.45	0.501	0.616	0.985	Agathobacter sp.
ASV302	6.25	1.19	2.49	0.477	0.634	0.985	Clostridia UCG-014 sp.
ASV309	27.3	1.49	2.01	0.745	0.456	0.985	Oscillospirales sp.
ASV310	3.09	-3.21	2.53	-1.27	0.206	0.985	Lactobacillus sp.
ASV311	8.3	0.826	0.705	1.17	0.242	0.985	UBA1819 sp.
ASV315	2.92	-2.71	2.95	-0.917	0.359	0.985	Oscillospirales sp.
ASV320	6.38	3.97	2.95	1.35	0.177	0.985	Clostridia UCG-014 sp.
ASV321	2.11	-4.14	2.96	-1.4	0.162	0.985	Peptococcus sp.
ASV322	1.94	3.45	2.95	1.17	0.242	0.985	Bilophila wadsworthia

ASV323	1.69	-3.44	2.96	-1.16	0.245	0.985	Bifidobacterium sp.
ASV328	7.52	-0.437	1.78	-0.246	0.806	0.985	Clostridia UCG-014 sp.
ASV330	0.929	-1.38	2.96	-0.465	0.642	0.985	Lachnospiraceae sp.
ASV331	1.21	-3.36	2.96	-1.14	0.256	0.985	Oscillospirales sp.
ASV332	0.36	-0.655	2.97	-0.221	0.825	0.985	Lachnospiraceae sp.
ASV337	0.0764	0.68	2.97	0.229	0.819	0.985	Bacteroides sp.
ASV339	9.27	0.197	1.2	0.164	0.87	0.985	Lachnospiraceae sp.
ASV341	16.4	-0.384	1.16	-0.33	0.741	0.985	Howardella ureilytica
ASV345	1.36	4.31	2.95	1.46	0.145	0.985	Dialister succinatiphilus
ASV348	3.17	-1.93	2.95	-0.655	0.513	0.985	Blautia sp.
ASV351	0.648	-2.52	2.96	-0.851	0.395	0.985	Catenibacterium sp.
ASV352	9.22	-0.513	0.806	-0.636	0.525	0.985	Incertae Sedis sp.
ASV353	1.95	-4.03	2.96	-1.36	0.173	0.985	Subdoligranulum sp.
ASV354	3.95	1.28	2.44	0.523	0.601	0.985	Clostridia sp.
ASV356	3.7	-0.626	2.94	-0.213	0.832	0.985	Blautia luti
ASV359	0.573	1.73	2.96	0.584	0.559	0.985	Clostridia UCG-014 sp.
ASV360	0.5	-2.2	2.96	-0.741	0.458	0.985	Blautia sp.
ASV363	5.08	0.182	2.67	0.0682	0.946	0.985	Lachnospiraceae sp.
ASV373	26.1	-0.178	0.93	-0.191	0.849	0.985	Incertae Sedis sp.
ASV374	4.41	-0.493	2.28	-0.217	0.829	0.985	Subdoligranulum sp.
ASV375	3.8	-1.03	2.44	-0.423	0.673	0.985	Bacteroides clarus

ASV379	5.01	0.712	1.43	0.499	0.618	0.985	Coriobacteriales Incertae Sedis sp.
ASV380	0.65	-2.52	2.96	-0.85	0.396	0.985	Prevotella sp.
ASV399	13.7	0.68	0.918	0.74	0.459	0.985	Alistipes ihumii
ASV409	0.751	-2.71	2.96	-0.916	0.36	0.985	Clostridia UCG-014 sp.
ASV412	0.517	-2.23	2.96	-0.754	0.451	0.985	Bilophila sp.
ASV413	1.56	0.339	2.95	0.115	0.909	0.985	Faecalibacterium sp.
ASV415	1.3	-3.46	2.96	-1.17	0.242	0.985	Clostridium sensu stricto 1 sp.
ASV416	7.26	-0.519	0.869	-0.597	0.55	0.985	Oscillibacter sp.
ASV422	1.26	-1.25	2.13	-0.586	0.558	0.985	Lactobacillus delbrueckii
ASV426	10	1.37	1.57	0.871	0.384	0.985	Clostridia UCG-014 sp.
ASV428	7.06	0.105	1.47	0.071	0.943	0.985	Lachnospiraceae sp.
ASV429	1.09	-3.22	2.96	-1.09	0.276	0.985	Ruminococcus sp.
ASV432	3.93	-2.2	2.85	-0.772	0.44	0.985	UCG-008 sp.
ASV437	11.9	1.46	1.19	1.23	0.22	0.985	Lachnospiraceae sp.
ASV441	0.527	-2.26	2.96	-0.763	0.446	0.985	Blautia glucerasea
ASV448	2.32	0.939	2.59	0.362	0.717	0.985	Clostridia UCG-014 sp.
ASV464	7.32	-0.148	1.16	-0.128	0.898	0.985	Slackia isoflavoniconvertens
ASV481	8.51	1.08	1.63	0.663	0.507	0.985	Lachnospiraceae sp.
ASV483	6.33	-0.132	1.51	-0.0872	0.93	0.985	Eggerthella lenta
ASV492	0.407	-1.94	2.96	-0.655	0.512	0.985	Agathobacter sp.
ASV528	2.16	0.183	2.38	0.0769	0.939	0.985	Alistipes massiliensis
ASV544	2.56	0.152	2.94	0.0515	0.959	0.985	Romboutsia sp.

ASV548	11.9	0.663	1.17	0.565	0.572	0.985	Christensenellaceae R-7 group sp.
ASV571	1.14	1.69	2.95	0.574	0.566	0.985	Clostridium sensu stricto 1 disporicum
ASV581	0.637	-0.294	2.96	-0.0992	0.921	0.985	Lactobacillus sp.
ASV626	0.897	-2.95	2.96	-0.998	0.318	0.985	Clostridium sensu stricto 1 tertium
ASV646	0.314	-1.49	2.96	-0.501	0.616	0.985	Lactobacillus sp.
ASV655	1.66	-3.81	2.96	-1.29	0.198	0.985	Clostridium sensu stricto 1 sp.
ASV660	8.8	-0.266	2.81	-0.0945	0.925	0.985	Leuconostoc sp.
ASV669	1.27	-3.43	2.96	-1.16	0.246	0.985	Lachnospiraceae sp.
ASV762	1.14	4.06	2.95	1.37	0.169	0.985	Agathobacter sp.
ASV813	0.0922	-0.227	2.97	-0.0766	0.939	0.985	Ruminococcus sp.
ASV867	0.128	-0.571	2.97	-0.193	0.847	0.985	Sarcina ventriculi
ASV1047	0.627	-2.47	2.96	-0.835	0.404	0.985	Agathobacter sp.
ASV48	109	-0.0888	2.09	-0.0425	0.966	0.986	Catenibacterium mitsuokai
ASV112	20.3	0.0688	1.48	0.0465	0.963	0.986	Oscillospirales sp.
ASV490	1.55	0.0775	2.95	0.0263	0.979	0.996	Lachnospiraceae sp.
ASV163	3	0.0144	2.94	0.00488	0.996	0.998	Lachnospiraceae sp.
ASV174	19.9	-0.00274	1.31	-0.00209	0.998	0.998	Lachnospira sp.
ASV317	10.4	0.00216	1.01	0.00214	0.998	0.998	Lachnospiraceae sp.
ASV402	11.7	-0.0335	2.4	-0.0139	0.989	0.998	Bacteroides ovatus
ASV566	0.047	-0.0165	2.97	-0.00557	0.996	0.998	Barnesiella sp.
